# The feasibility of ^11^C‐PIB‐PET/CT for amyloid plaque burden: validation of the effectiveness of CT‐based partial volume correction

**DOI:** 10.1002/brb3.532

**Published:** 2016-08-01

**Authors:** Kei Sasaki, Norihide Maikusa, Etsuko Imabayashi, Tetsuya Yuasa, Hiroshi Matsuda

**Affiliations:** ^1^ Graduate School of Science and EngineeringYamagata UniversityYamagataJapan; ^2^ Integrative Brain Imaging CenterNational Center of Neurology and PsychiatryKodairaTokyoJapan; ^3^ Department of Nuclear MedicineSaitama Medical University International Medical CenterSaitamaJapan

**Keywords:** Alzheimer disease, amyloid plaques/RI, magnetic resonance imaging, multidetector computed tomography, positron‐emission tomography/MT

## Abstract

**Introduction:**

Amyloid positron‐emission tomography (PET) imaging with ^11^C‐Pittsburgh compound B (PiB) is an effective tool for assessing brain amyloid deposits. PET imaging, however, can suffer from the partial volume effect (PVE). PVE has been corrected using MRI (magnetic resonance imaging) image data. However, correction of the PVE of PET using MRI usually requires two separate procedures, imposing a burden on patients and leading to low throughput and inefficient diagnoses. The advent of PET/computed tomography (PET/CT) may potentially overcome these problems and offer higher throughput and reliable quantification of amyloid plaques and assessment of Alzheimer disease (AD).

**Methods:**

We investigated the feasibility of correcting PVE in amyloid PET using CT, obtained by PET/CT, instead of MRI. We demonstrated the efficacy of partial volume correction (PVC) based on CT by comparing the results of CT‐based PVC and those of MRI‐based PVC using images acquired from AD patients and controls.

**Results:**

Both methods were able to perform PVC. Slight but significant differences between standard uptake volume ratio (SUVR) values were noted between the two modalities; these were attenuated by constant multiplication.

**Conclusion:**

CT will potentially replace MRI for PVC, allowing the use of a single PET/CT scanner for amyloid plaque quantitation.

## 1 Introduction

Misfolded amyloid beta (Aβ) proteins accumulate in the brains of Alzheimer disease (AD) patients (Zhang et al., 2004). Amyloid imaging using positron‐emission tomography (PET) can determine the distribution of Aβ accumulation in the cerebral cortex using the radioactive tracer, ^11^C‐PiB (Pittsburgh compound B; Brockschnieder et al., 2012). The specific accumulation of Aβ in gray matter (GM) causes it to have a higher ^11^C‐PiB signal intensity than white matter (WM) in PET images of AD patients, whereas in healthy controls, only nonspecific accumulation in WM is observed (Klunk et al., 2004). PET amyloid imaging can be an effective diagnostic aid for early‐stage AD, which can be difficult to detect with the existing diagnostic methods (Braak & Braak, 1991; Drzezga et al., 2008; Dubois et al., 2010; Förster et al., 2012; Hirata et al., 2005; Ikonomovic et al., 2008; Klunk et al., 2004; Lowe et al., 2009; Ma et al., 2014; Mikhno et al., 2008; Price et al., 2005; Rabinovici et al., 2008; Wolk et al., 2009; Zhang et al., 2014).

One of the limitations of PET amyloid imaging is the partial volume effect (PVE), which are caused by the two distinct phenomena, that is, the finite resolution effect and the tissue fraction effect. The former effect is image blurring introduced by the insufficient spatial resolution of PET scanner, leading to spill‐in and spill‐out effects between regions. The latter effect occurs within voxels at the boundaries of different tissue types and can lead to the mismeasurement of tracer uptake because the uptakes of the two or more different tissues in a voxel are averaged. When using ^18^F‐FDG‐PET to detect a decrease in glucose accumulation, PVE often causes an overestimation of the hypometabolism, resulting in acceptance of abnormal findings. By contrast, in PET amyloid imaging, PVE may cause an underestimation of abnormal uptake; consequently, abnormal findings may be overlooked. Therefore, partial volume correction (PVC) for PET amyloid imaging is needed for reliable quantitative evaluation.

For PVC, the brain image needs to be segmented into GM, WM, and CSF (cerebrospinal fluid) regions. Many methods for this have been reported (Giovacchini et al., 2004; Kato et al., 2008; Matsuda et al., 2003; Muller‐Gartner et al., 1992; Quarantelli et al., 2004; Shidahara et al., 2009; Thomas et al., 2011), usually involving MR image data. Some decisive algorithms that stably segment an MRI brain image have been developed (such as that of Schöder et al.) and in many cases, PVC using the MR image's segmented anatomical information improves diagnostic accuracy. However, to use this PVC, both PET and MRI must be performed, usually as separate procedures (although PET/MRI is not yet widely available), leading to reduced efficiency and burdening the patient. Moreover, MRI has insufficient reproducibility between intersite and/or intermachine procedures, producing artifacts caused by the inhomogeneity of the magnetic field (Maikusa et al., 2013), for example, inhomogeneous signal intensity (Sled, Zijdenbos, & Evans, 1998) and geometric distortion. Postprocessing steps must be applied to correct for these effects.

In recent years, PET/CT has been developed to not only correct for the effect of tissue gamma ray attenuation on PET but to also compensate for PET's limited resolution (Ciernik et al., 2003; Schöder, Erdi, Larson, & Yeung, 2003). Because CT and PET scanning are performed in rapid succession in PET/CT, the process is efficient, and misregistration between PET and CT images is avoidable. In addition, CT is less susceptible to intermachine differences, thereby ensuring the reproducibility of results between machines and sites. With PET/CT available for PET amyloid imaging, if CT can be used for PVC instead of MRI, the efficiency of amyloid quantification would be dramatically improved while maintaining evaluation accuracy. Imabayashi et al. (2013) have reported that the voxel‐based morphology (VBM) technique could be performed based on CT obtained from amyloid PET/CT instead of MRI using statistical parametric mapping 8 (SPM 8). Thereby, it was demonstrated that GM could be successfully segmented from CT using SPM 8 as well.

The purpose of this research was to examine the possibility of using the CT image from the PET/CT scanner for PVC of the ^11^C‐PiB‐PET image and to investigate whether PVC can improve its accuracy in detecting amyloid deposits, and to compare it with that of MRI‐based PVC.

## 2 Materials and Methods

### 2.1 Subjects

We assessed nine AD patients (two women and seven men, 74.9 ± 5.8 years old) who were diagnosed with AD according to the National Institute of Neurologic and Communicative Disorders and Stroke and the Alzheimer's Disease and Related Disorders Association criteria, and 11 cognitively normal controls (four women and seven men, 67.5 ± 3.8 years old) who had no unusual accumulation of PiB. ^11^C‐PiB‐PET/CT and MRI scans were performed on all subjects within a mean interval of 25.1 ± 8.2 days (14–40 days) in the Department of Nuclear Medicine, Saitama Medical University International Medical Center or in the National Center of Neurology and Psychiatry. The ethics committees of both Saitama Medical University and National Center of Neurology and Psychiatry approved this study, and all subjects gave written informed consent to participate.

### 2.2 Image acquisition

For PET/CT, (Biograph 6 Hi‐Rez^®^, Siemens Medical Solutions, Inc., Knoxville TN, USA) the PET data was composed of 81 sections of 168 × 168 pixels; the detective element size was 2.03 × 2.03 mm^2^; the section thickness was 2.00 mm. The 25 PET images per subject were reconstructed by applying FORE + OSEM to the list‐mode data acquired 70 min after an intravenous injection of 600 MBq of 2‐[4′‐(methylamino)phenyl]‐6‐hydroxybenzo‐thiazole (^11^C‐PiB). From these volumes, a PET image was generated by averaging the last four volumes, which were acquired at 5‐min intervals from 50 to 70 min after injection, and processed with a three‐dimensional Gaussian filter with a standard deviation (*SD*) of 5.0 × 5.0 × 5.0 mm^3^.

Before the intravenous injection, a helical CT scan was performed using the same PET/CT scanner. The CT image was composed of 109 sections of 512 × 512 pixels; the detective element size was 0.49 × 0.49 mm^2^; the section thickness was 3.00 mm. The scanning parameters were as follows: 1.0 s gantry rotation time, 130 kVp, 240 mAs, 0.5:1 beam pitch, 3‐mm table feed per gantry rotation, 6 × 2 detector configuration. The image was reconstructed by the filtered back projection method.

A T1‐weighted MR image was obtained using a 1.5 T scanner (Symphony^®^, Siemens, Inc.) using magnetization‐prepared rapid gradient‐echo (MP‐RAGE) technique under the following conditions: the repetition time (TR), inversion time (TI), and flip angle were 2,400 or 3,000 ms, 1,000 ms, and 8°, respectively. The MR image was composed of 160 sections of 192 × 192 pixels; the in‐plane spatial resolution was 1.25 × 1.25 mm^2^; the section thickness was 1.20 mm, and the FOV was 240 × 240 mm^2^. After reconstruction, the geometric distortion correction proposed by Maikusa et al. (2013) and a correction for inhomogeneous signal intensity (Jack et al., 2008) were applied.

### 2.3 Image processing

**Figure 1:**
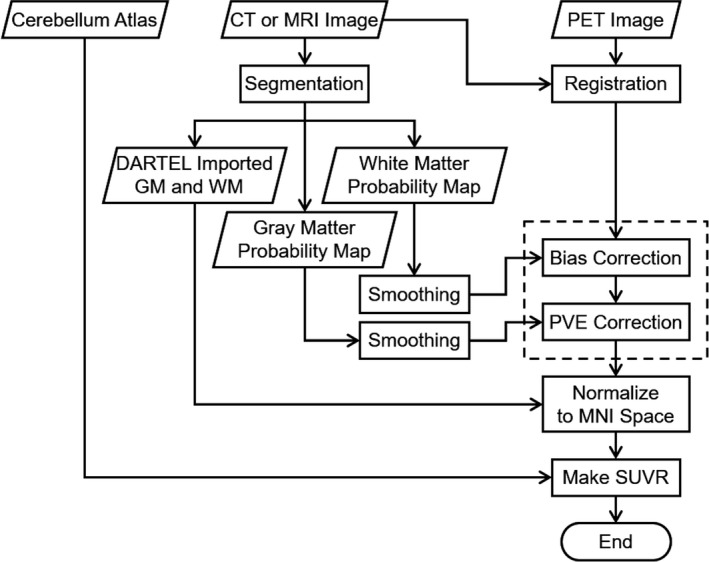
Flowchart of the processing. The steps surrounded by a dotted box correspond to partial volume correction (PVC)

The flow of the proposed processing is shown in Fig. 1. The analysis was performed using a macro program in SPM 8 created using commercial software (MATLAB R2014a^®^, Mathworks Inc., Natick MA, USA). The registration between the MRI and PET images was carried out using the process “Coregister: Estimate” prepared in SPM 8.

### 2.4 Segmentation

The MRI and CT images were segmented into GM and WM regions before PVC. We applied the process “New Segment”, prepared in SPM 8, for tissue segmentation. Because it is relatively difficult to segment the CT image with high reliability owing to its low contrast in brain tissue, we carefully selected the setting parameters and options in “New Segment”. We set “Bias Regulation” and “Bias FWHM” to “No Regulation” and “No Correction”, respectively, because the CT image has a homogenous signal. In addition, “Num. Gaussians” was set to “Nonparametric”. The parameters were set to default for MRI segmentation.

“New Segment” takes the CT or MRI image and outputs GM and WM probability maps, which are employed in the PVC processes explained below. It also outputs the GM and WM DARTEL‐imported probability maps, which were imported into Diffeomorphic Anatomical Registration Through Exponential Lie Algebra (DARTEL; Payoux et al., 2015) to transform each image into standardized space, as described in section below. We applied a three‐dimensional Gaussian filter with a *SD* of 8.0 × 8.0 × 8.0 mm^3^ to the respective probability maps from “New Segment” to match their spatial resolution with that of the PET image. The *SD* of Gaussian filter was decided for the following reason: at first, we assumed that the spatial resolution of PET and CT scanners were prescribed by the Gaussian point spread function (PSF). In addition, we assumed that the *SD* of PSF for CT and PET scanners were 1.0 and 4.0 mm, although their detective element size were 0.49 × 0.49 and 2.03 × 2.03 mm^2^, respectively. As we applied the Gaussian filter with a *SD* of 5.0 mm when creating the PET image, the *SD* of PSF of the resultant PET image was 9.0 (=4.0 + 5.0) mm because of the reproductive property of the Gaussian function. Therefore, under the assumption that the *SD* of PSF of the probability maps are the same as that of CT, the discrepancy of *SD* between PET and probability maps is 8.0 (=9.0−1.0) mm.

### 2.5 Partial volume correction

PVC was performed using the smoothed GM and WM probability maps using the algorithm shown in the dotted box in Fig. 1; the fundamental concept for this approach was devised by Matsuda et al. (2003) for MRI‐based PVC of SPECT.

Generally, the PiB PET image is biased due to nonspecific accumulation, which degrades the evaluation accuracy of amyloid deposits. We corrected the bias as follows: first, we generated a WM mask by applying a threshold of 95% to the smoothed WM probability map and masked the PET image with it. Next, we generated the median value of the masked PET image. For each voxel, we declared the product of the median and the voxel value of the corresponding voxel in the smoothed WM probability map as the bias signal, and then subtracted the product from the voxel value of the PET image. Matsuda et al. (2003) used the maximum, instead of the median, as a representative value of the WM region, whereas we adopted the median because outliers may affect subsequent processing.

For each voxel of the bias‐corrected PET image, the final voxel value was found by dividing the corrected PET voxel value by the value of the corresponding voxel of the smoothed GM probability map.

Finally, we generated a GM mask by applying a threshold of 35% to the smoothed GM probability map and masked the PVE‐corrected PET image with it to exclude regions irrelevant to evaluation of amyloid deposits. Below, the resultant image is referred to as the “PVC‐PET image”.

### 2.6 Calculation of the SUVR

Currently, the SUVR is the most widely used quantitative method in PiB PET because it requires neither a dynamic scan nor blood sampling. To create an SUVR image, the cerebellar cortex is often used as a reference because it has almost no specific accumulation of ^11^C‐PiB. After using DARTEL to transform the CT or MR image into standardized space or MNI (Montreal Neurological Institute) space, we transformed the PVC‐PET image into MNI space using the same transformation. Finally, we located the cerebellar cortex by marking it as a volume of interest (VOI) on the transformed PVC‐PET image.

The details of the analysis are as follows: the default settings of DARTEL were used. The inputs were 20 GM DARTEL probability maps and 20 WM DARTEL probability maps, obtained from “New Segment” for all subjects. The resulting DARTEL template and flow field output were used to transform the PVC‐PET image to MNI space.

**Figure 2:**
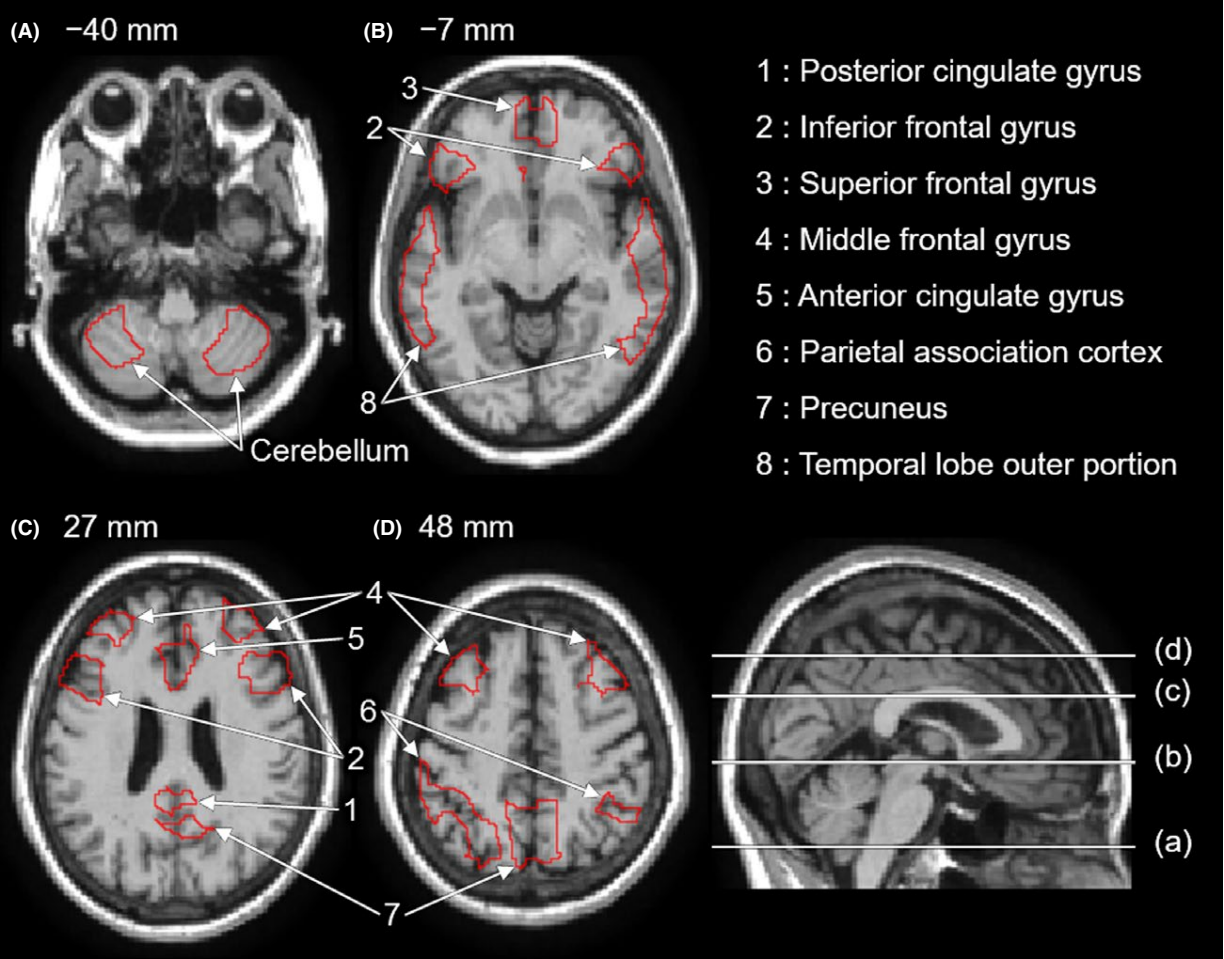
Two volumes of interest (VOIs) located in the cerebellar cortex (A) and eight VOIs for Alzheimer disease (AD) assessment (B, C, and D), generated by restricting the regions prepared in the AAL (automated anatomical labeling) atlas to the corresponding central regions by erosion. The VOIs are surrounded by red curves

The VOI was generated with respect to the cerebellar cortex and was prepared using the automated anatomical labeling (AAL) atlas (Tzourio‐Mazoyer et al., 2002). Because the cerebellar cortex has small foliae at the periphery, and only the central region can be transformed into MNI space using DARTEL with high reliability, using the cerebellar cortex without correction decreases the accuracy of estimating the reference value. Instead, we restricted analysis to the central cortical region using erosion, a type of morphological filter, such that the number of voxels in the eroded region was reduced to half the original number. The VOI obtained is shown in Fig. 2A.

We used the median value of the VOI within the PVC‐PET image that was transformed into MNI space as the reference value for SUVR creation. Finally, we obtained the SUVR image by dividing the value in each voxel of the PVC‐PET image by the reference value of the cerebellar cortex. Below, we refer to the SUVRs with CT‐ and MRI‐based PVC as the “CT‐SUVR” and “MRI‐SUVR”, respectively.

### 2.7 Positioning VOIs for AD assessment

We generated the VOIs for amyloid assessment based on the regions prepared in the AAL atlas, in which the posterior cingulate gyrus, inferior frontal gyrus, superior frontal gyrus, middle frontal gyrus, anterior cingulate gyrus, parietal association cortex, precuneus, and the outer portion of the temporal lobe are considered. Similar to the cerebellar cortex, we restricted each region to the eroded central regions of each VOI, such that the number of voxels in the eroded region was reduced to half the original number of voxels. The VOIs obtained are shown in Fig. 2B and C.

### 2.8 Statistical analysis

We investigated whether the results of CT‐based PVC correlated with those of MRI‐based PVC. Using R software (R Project for Statistical Computing, RRID:SCR_001905), we plotted the MRI‐SUVR values against the CT‐SUVR values of the individual voxels in each VOI for all subjects and obtained a regression line and slope. Next, assuming that the mean α of the slopes obtained from the individual VOIs is applicable to other regions throughout the GM, we created an image by multiplying each voxel value in the CT‐SUVR image by α, the product of which is referred to as the “α‐corrected CT‐SUVR”.

We evaluated the differences between α‐corrected CT‐SUVR values and MRI‐SUVR values in the AD and NL groups using a *t* test based on a general linear model with a significance threshold of *p *<* *.001, which was conducted offline using SPM 8.

For comparing the values of CT‐SUVR, MRI‐SUVR, and SUVR without PVC in the AD and NL groups in each VOI, we analyzed the distributions of the voxel values and displayed the results with boxplots. The differences between the two groups were evaluated by a Mann–Whitney *U* test with a significance threshold of *p *<* *.005, which was conducted offline using R.

## 3 Results

The processing time for creation of the CT‐SUVR images for 20 subjects was about 3 hr (~9 min per subject). We used a processor (Core i7‐3820^®^, Intel, Santa Clara CA, USA) at 3.6 GHz that had 32 GB of installed memory (RAM) and ran on 64‐bit software (Windows 7^®^, Microsoft, Inc., Redmond WA, USA).

### 3.1 Segmentation

**Figure 3:**
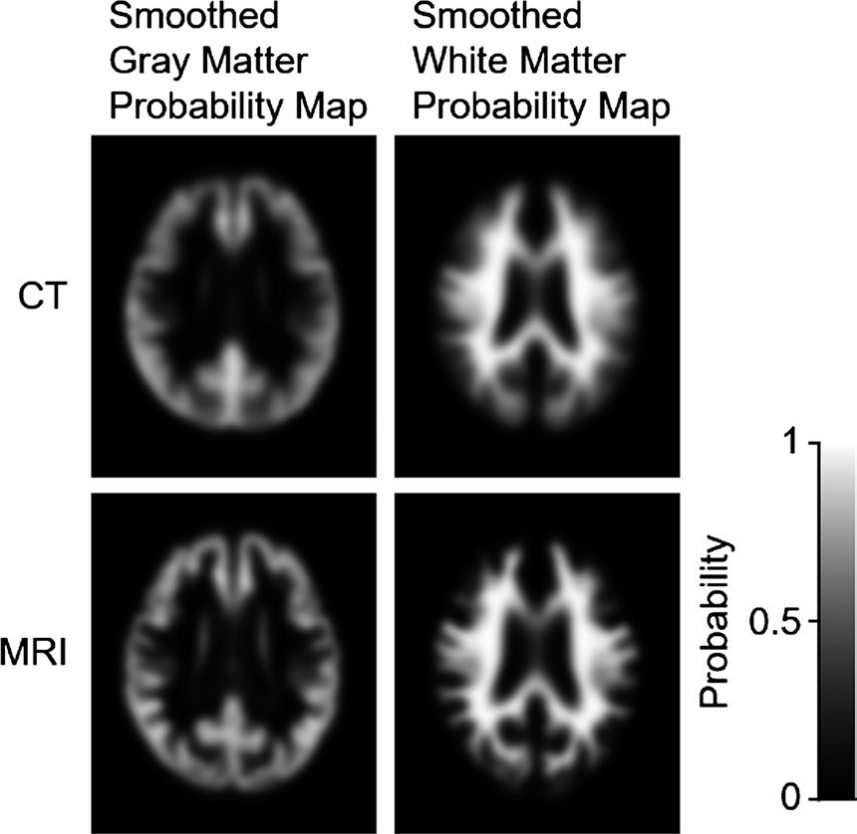
Examples of the smoothed probability maps obtained from the CT and MR image of a single subject. Top left: GM from CT; top right: WM from CT; bottom left: GM from MRI; and bottom right: WM from MRI

Examples of the smoothed GM and WM probability maps obtained from the CT and MRI images for a single subject are shown in Fig. 3. The smoothed GM and WM probability maps from CT were noisier than those from MRI, especially for the gyri.

### 3.2 SUVR with PVC

**Figure 4:**
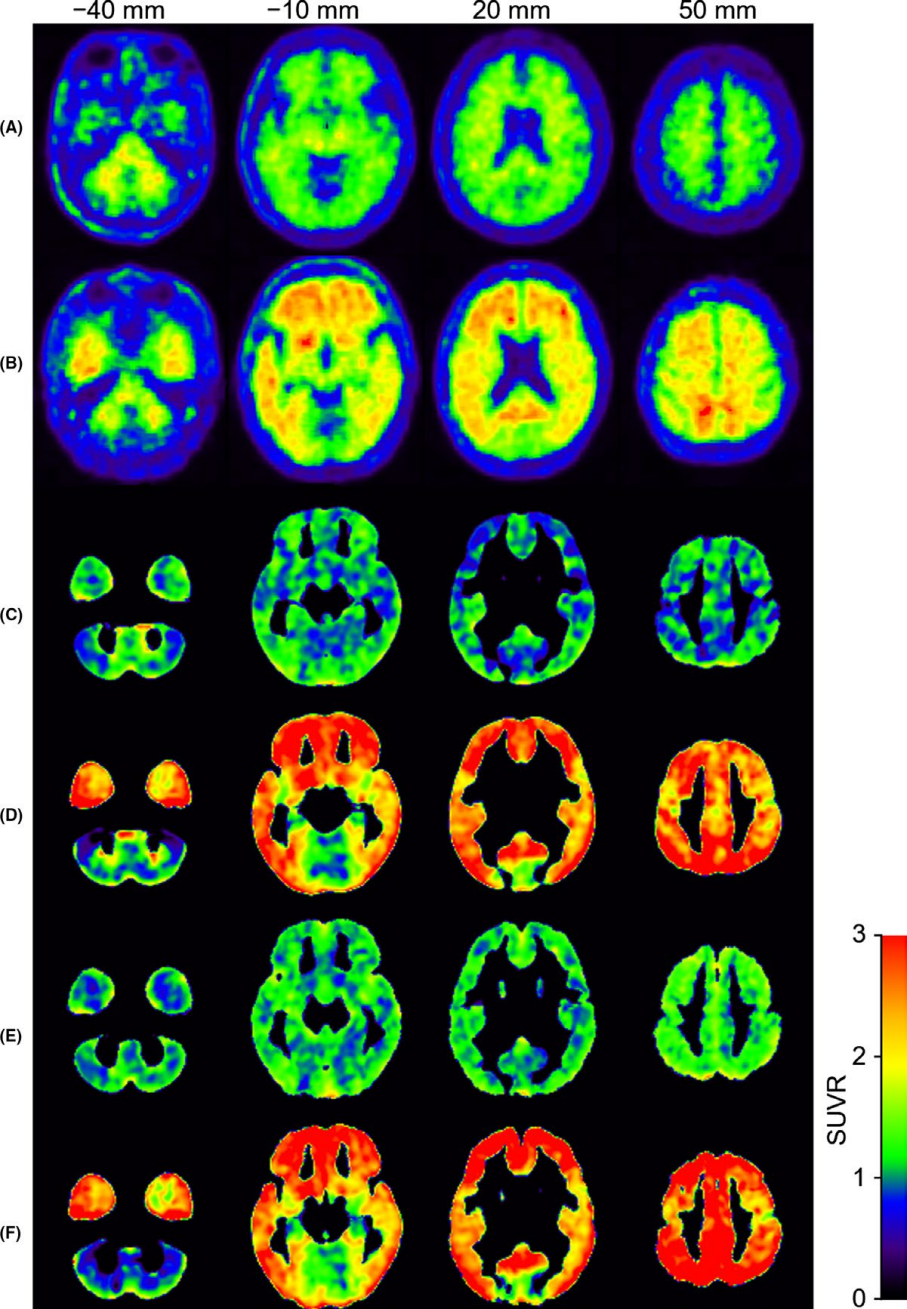
Examples of the SUVR cross‐sectional images generated from a normal volunteer (rows A, C, and E) and an Alzheimer disease (AD) patient (rows B, D, and F). Rows A and B correspond to standard uptake volume ratios (SUVRs) without partial volume correction (PVC). Rows C and D correspond to SUVRs with CT‐based PVC. Rows E and F correspond to SUVR with MRI‐based PVC. The columns, left to right, correspond to axial images at the levels of −40, −10, 20, and 50 mm, respectively. The signal intensities range from 0.0 to 3.0

SUVR images for a normal volunteer and an AD patient are shown in Fig. 4. The nonspecific accumulation in the WM of normal volunteers is the bias (Fig. 4A). The signal intensity within the GM for AD patients (Fig. 4B, D and F) was higher than those of the normal volunteers (Fig. 4A, C and E), regardless of whether PVC was applied. In the CT‐ and MRI‐SUVR images for AD patients (Fig. 4D and F, respectively), the signal intensity was relatively high throughout the GM, as compared to the SUVR image without PVC (Fig. 4B). The SUVR values with MRI‐based PVC appeared to be slightly higher than those with CT‐based PVC in both the controls and patients (Fig. 4C, D, E and F).

**Figure 5:**
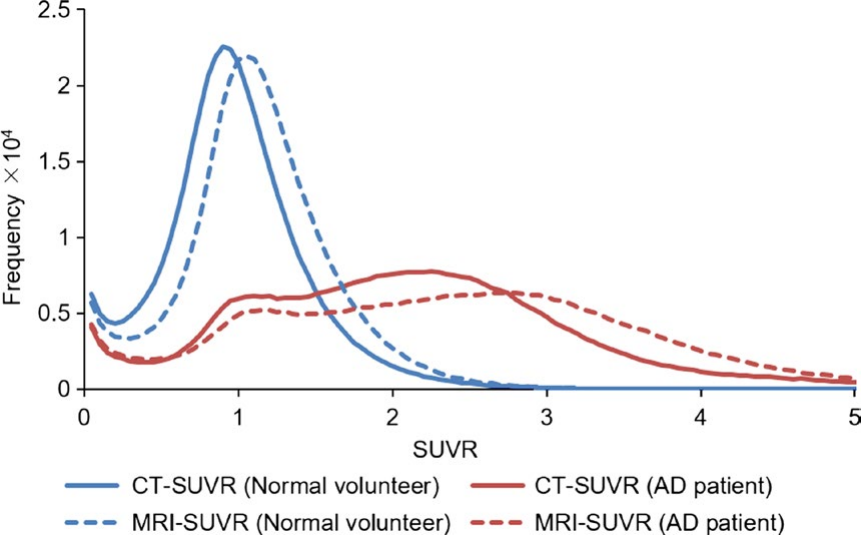
The histograms of the CT standard uptake volume ratio (SUVR) and MRI‐SUVR values for the Alzheimer disease (AD) and normal (NL) groups. Blue solid line, red solid line, blue dotted line, and red dotted line correspond to CT‐SUVR of the NL group, CT‐SUVR of the AD group, MRI‐SUVR of the NL group, and MRI‐SUVR of the AD group, respectively

Histograms of the GM voxel values obtained from the CT‐ and MRI‐SUVR images are shown in Fig. 5 and led to the following observations:

1. For both CT‐ and MRI‐SUVRs, the range of values for the AD group was wider than that of the values of the NL group.
2. For both NL and AD groups, the values from MRI‐SUVR were shifted to the right, compared with those from CT‐SUVR, indicating that the voxel values of MRI‐SUVR tended to be higher than those of CT‐SUVR, as exemplified in Fig. 4C–F.

The observation that the AD group had a wider range of SUVR values reflects the fact that the AD group had nonspecific accumulation around the cerebellar cortex and specific accumulation in GM (Fig. 4D and F). The observation of the narrower range of SUVR values in normal volunteers reflects the fact that the NL group had nonspecific accumulation in GM, resulting in most regions having a SUVR value near 1.0 (Fig. 4C and E).

We found that the MRI‐SUVR values tended to be higher than the CT‐SUVR values in both the NL and AD groups, although the histogram shape was similar. The shift is closely related to the fact that because the CT image has lower soft tissue contrast, the GM region is wider or coarser than that of the MR image after tissue segmentation (Fig. 3).

### 3.3 Correlation between CT‐SUVR and MRI‐SUVR

**Figure 6:**
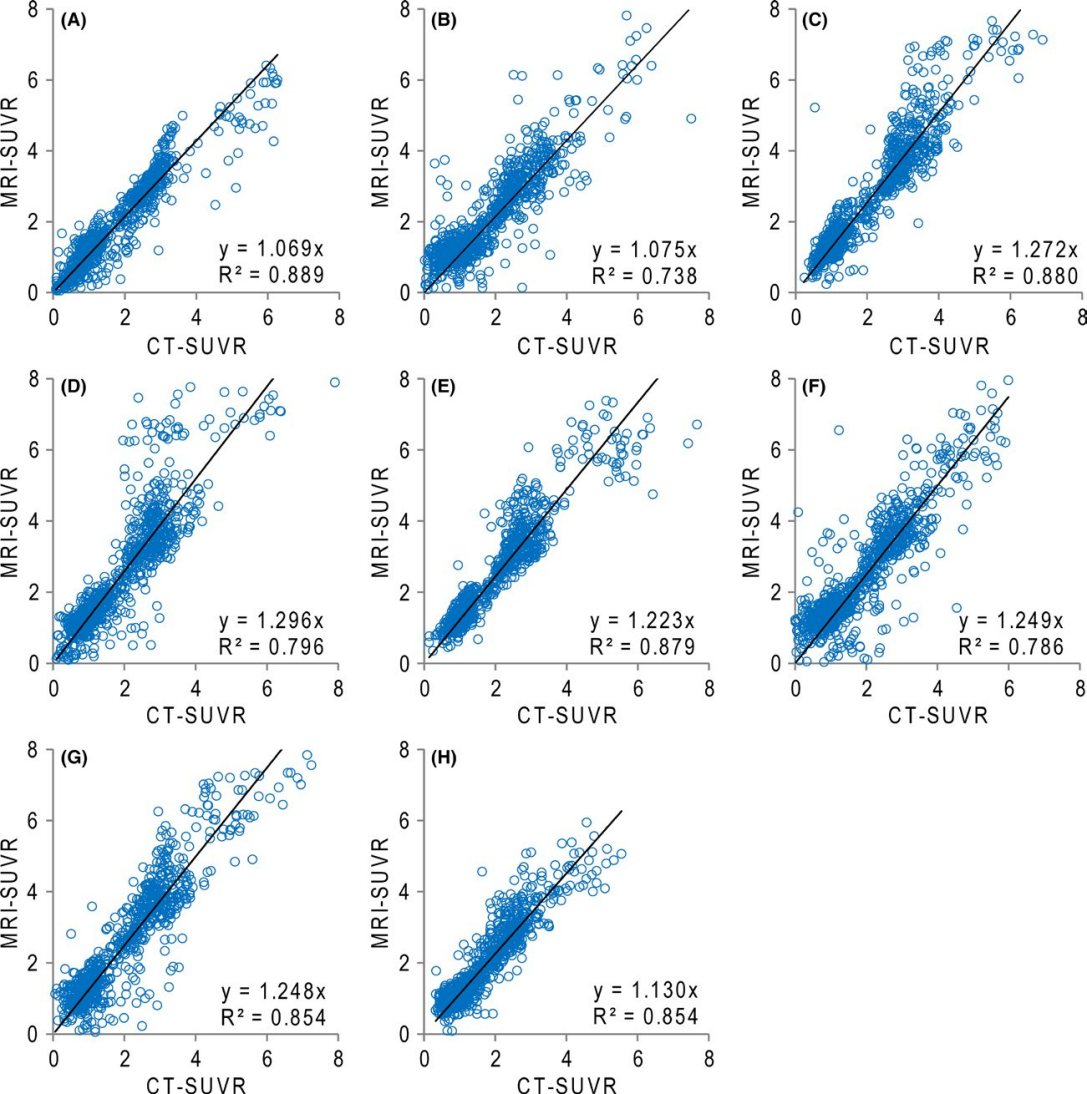
Correlation between the CT standard uptake volume ratio (SUVR) and MRI‐SUVR values of each volume of interest (VOI). (A) posterior cingulate gyrus, (B) inferior frontal gyrus, (C) superior frontal gyrus, (D) middle frontal gyrus, (E) anterior cingulate gyrus, (F) parietal association cortex, (G) precuneus, and (H) temporal lobe, outer portion

The scatter plots of the MRI‐SUVR values against the CT‐SUVR values for the individual VOIs are shown in Fig. 6. In this figure, we randomly selected a representative sample of 1,000 voxels in each VOI to make it easier to see. We found a strong correlation within the individual VOIs; all correlation coefficients were >.73. The slopes of the regression lines were >1.0, which supports 2. The average slope α was 1.192 ± 0.027.

**Figure 7:**
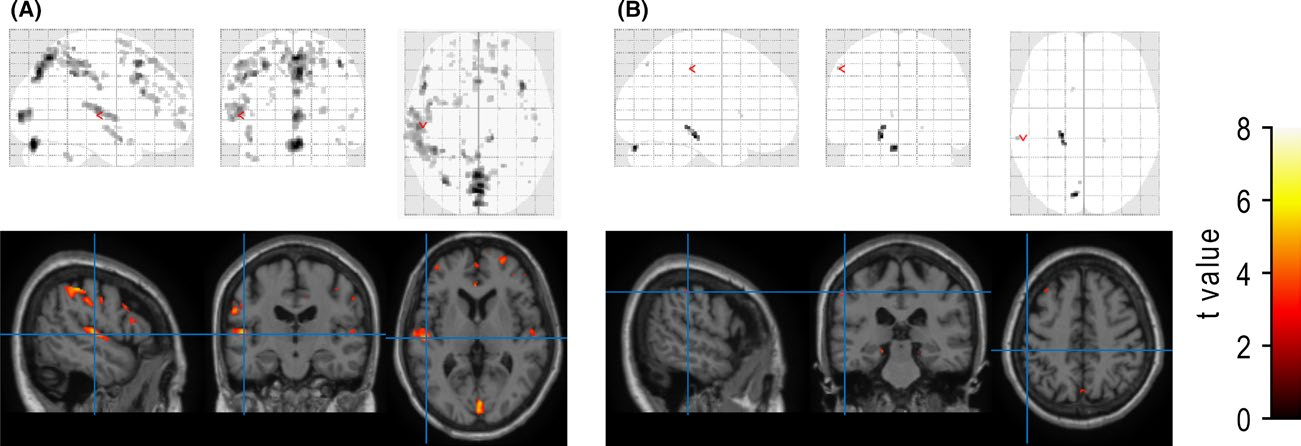
Results of the *t* test between the α‐corrected CT standard uptake volume ratio (SUVR) and MRI‐SUVR values for each voxel of the normal (NL) group (A) and Alzheimer disease (AD) group (B). In each part, the top and bottom rows correspond to the maximum intensity projection (MIP) representation and the spatial distribution of the *t* value, with a superimposed standardized brain cross section. The columns correspond to sagittal, coronal, and axial images, respectively

The results of the *t* test based on the general linear model between α‐corrected CT‐SUVR values and MRI‐SUVR values for the AD and NL groups are shown in Fig. 7A and B, respectively. We found no significant differences throughout the GM region for the AD group, whereas a significant difference was found in the gyri of the NL group.

As previously mentioned, the MRI‐SUVR values were higher than the CT‐SUVR values. However, for both the NL and AD groups, the histograms of the values from CT‐SUVR and MRI‐SUVR had similar shapes (Fig. 5). In addition, there was a strong correlation between the CT‐SUVR and MRI‐SUVR values for all VOIs (Fig. 6). This is further supported by the lack of significant differences between the α‐corrected CT‐SUVR values and MRI‐SUVR values for both groups in almost all VOIs. We conclude that the results of the CT‐ and MRI‐based PVC are compatible with each other, after the multiplication of a correction factor.

### 3.4 VOI analysis

**Figure 8:**
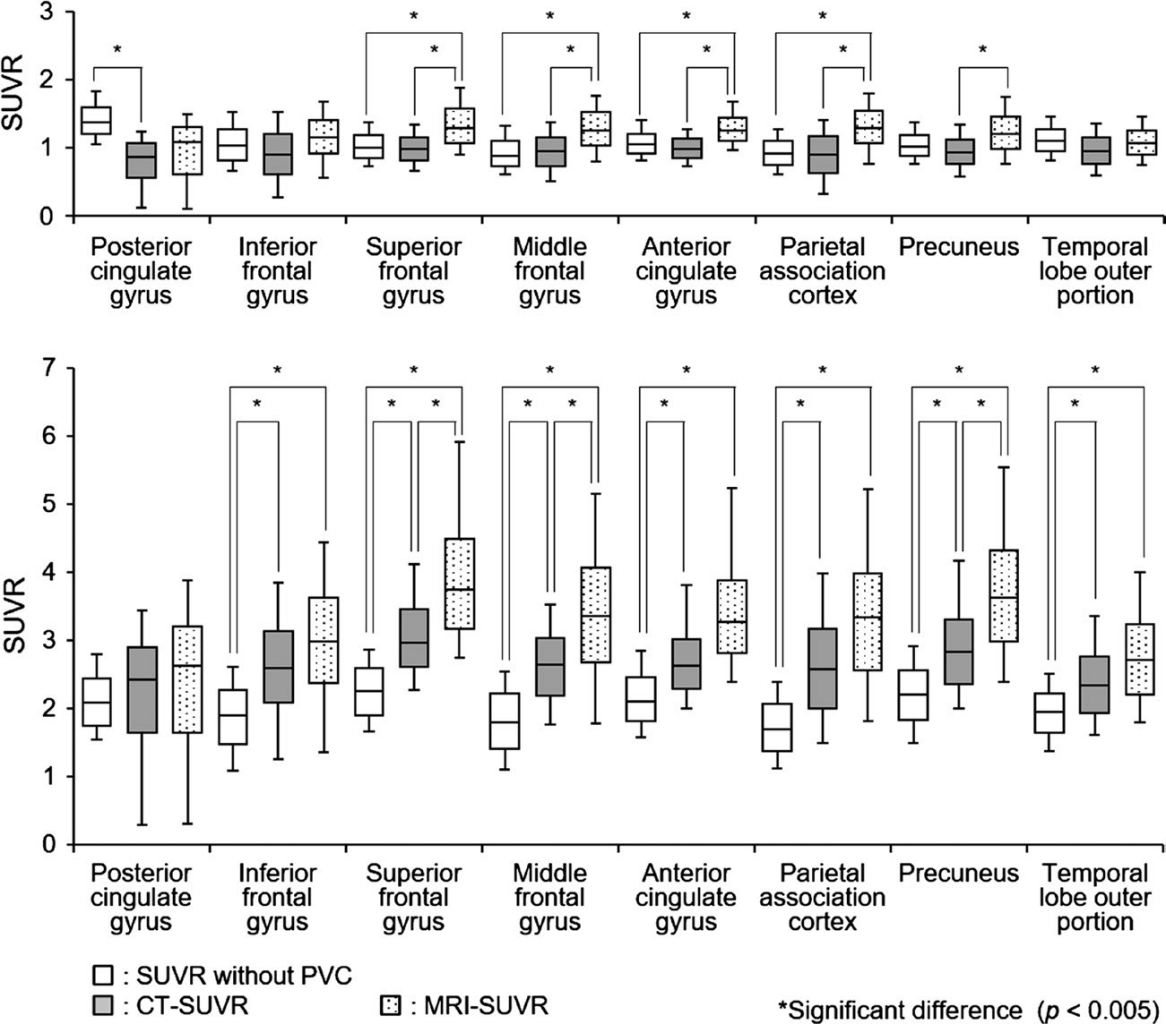
Distributions of standard uptake volume ratio (SUVR) without partial volume correction (PVC), CT‐SUVR, and MRI‐SUVR for the normal (NL) group (top) and the Alzheimer disease (AD) group (bottom) for each volume of interest (VOI). The left, middle, and right boxplots for each VOI correspond to SUVR without PVC, CT‐SUVR, and MRI‐SUVR, respectively. *represents a significant difference of *p *<* *.005

The distributions of values from CT‐SUVR, MRI‐SUVR, and SUVR without PVC in all the VOIs for the NL and AD groups are shown in Fig. 8. We confirmed that there was a significant difference between the AD and NL groups (*p *<* *.005) in all the VOIs, regardless of whether PVC was applied. We found that:

1. PVC increased the difference between the medians of the AD and NL groups.
2. PVC increased the difference between the first quartile of the AD group and the third quartile of the NL group, meaning less overlap between the distributions.

The observation that PVC increases the difference between the medians of the AD and NL groups and that PVC decreases the overlap of values demonstrates that PVC can further distinguish between the AD and NL groups.

The significant difference between the values of MRI‐SUVR and CT‐SUVR observed in five VOIs (superior frontal gyrus, middle frontal gyrus, anterior cingulate gyrus, parietal association cortex, and precuneus) of the NL group, and three VOIs (superior frontal gyrus, middle frontal gyrus, and precuneus) of the AD group correlates with the observation that the distribution of MRI‐SUVR values was shifted to the right in all GM regions.

**Figure 9:**
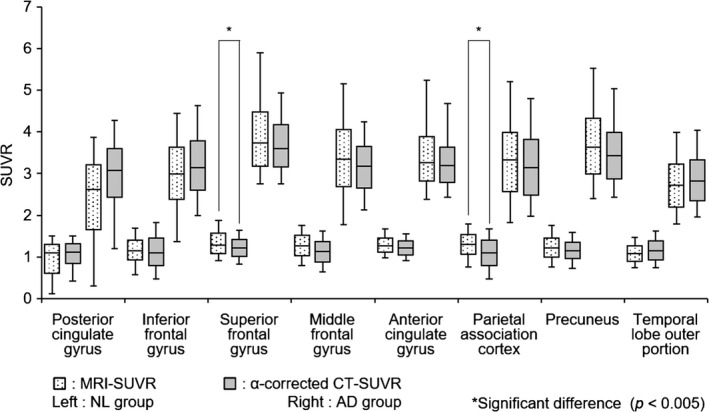
Distributions of the MRI standard uptake volume ratio (SUVR) and the α‐corrected CT‐SUVR values for the normal (NL) and Alzheimer disease (AD) groups for each volume of interest (VOI). In each VOI, the boxplots from left to right correspond to the MRI‐SUVR of the NL group, the α‐corrected CT‐SUVR of the NL group, the MRI‐SUVR of the AD group, and the α‐corrected CT‐SUVR of the AD group, respectively. The MRI‐SUVR values are the same as those in Fig. 8. * represents a significant difference of *p *<* *.005

A comparison of the distributions of α‐corrected CT‐SUVR and MRI‐SUVR in each VOI is shown in Fig. 9. Two VOIs of the NL group were significantly different (superior frontal gyrus and parietal association cortex), but the superior frontal gyrus, middle frontal gyrus, anterior cingulate gyrus, parietal association cortex, and precuneus were significantly different when the CT‐SUVR values were not corrected.

## 4 Discussion

The feasibility of PVC for PET based on CT and its efficacy have been investigated, which was motivated by the fact that PET/CT scanners are becoming widely available in the clinical setting, improving efficiency, and mitigating the burden on patients because the two separate scans are performed in the same apparatus in one sitting.

Because the PVC in this study was based on the probability maps of GM, WM, and CSF segmented from a structural image (MRI hitherto or CT in this study) using SPM 8, the effect of PVC seems to depend on the accuracy of probability maps. CT is inferior to MRI in segmentation accuracy because of its low soft tissue contrast, which makes CT segmentation less accurate in the detection of subtle structural change such as cortical thinning due to atrophy for evaluating disease progression. However, the segmented images were smoothed in the course of the PVC procedure using a Gaussian kernel with an *SD* of 8 mm (see Fig. 1) to equalize the resolution of the structural image with that of a PET image. Thus, it is conceivable that the final results do not depend on the differences in the accuracy of probability maps between MRI and CT. The smoothed probability maps from MRI and CT are quite similar, as shown in Fig. 3. This suggests that CT may be used for PVC instead of MRI.

As shown in Fig. 9, the constant multiplication enables matching the effects on VOI analysis by CT‐based PVC with those by MRI‐based PVC. In the NL group, the number of VOIs having a significant difference between MRI‐ and α‐corrected CT‐SUVR values was reduced from 5 to 2; even in the two VOIs with significant differences, the distributions of MRI‐ and CT‐SUVR were similar to each other as shown in Fig. 9. In the AD group, no VOIs having significant differences between CT and MRI were found. The fact that the results of CT‐based PVC are approximately equivalent to those of MRI‐based PVC in the NL and AD groups, despite the cortical thinning in AD patients, suggests that CT‐based PVC is effective for quantitation of amyloid burden in AD research for subjects ranging from AD through MCI to NL.

Generally in PET amyloid imaging, amyloid deposits are mainly evaluated by visual assessment by neuroradiologists. However, it has been reported that in amyloid PET, some equivocal findings are observed in interreading even among experienced neuroradiologists, and for such patients, SUVR tends to take intermediate values ranging from a typical value for AD to that for NL (Hosokawa et al., 2015; Payoux et al., 2015). The reason may be because PVE results in lower SUVR values despite sufficient amyloid deposits. It was shown from the results of this study that PVC can enhance specific amyloid accumulation regardless of the stage of cognitive impairment. PVC can be adapted to PET using other kinds of tracer. Hence, it is expected that our proposed PVC method will improve evaluation accuracy and will promote interreader agreement for equivocal findings.

It is known that there are cases where amyloid deposits are observed despite normal cognitive function, so‐called “preclinical AD”. However, the evolution process from normal and amyloid negative to preclinical AD has not been quantitatively observed in follow‐up surveys. With the proposed PVC, we can potentially trace the evolution from amyloid negative to amyloid positive with more reliability. Longitudinal research is important for elucidating the evolution of AD.

This study has limitations. First, the number of subjects included in this study was insufficient. The subjects included only AD and NL groups. Subjects with equivocal scans or ones showing specific accumulation despite normal cognitive function were not included. Presently, we are collecting follow‐up PET/CT data for subjects with a variety of scan appearances and a normal degree of cognitive function to combine cross‐sectional research with longitudinal research. We hope to demonstrate the efficacy of this PVC methodology in the evolution process. Second, the PVC has several parameters such as threshold level to determine GM or WM maps. These values were decided through trial and error. The values will depend on the imaging condition in PET and CT. We should standardize the parameters selection through the collective data. In addition, we should compare the proposed PVC with current state‐of‐the‐art PVC methodologies with fewer parameters (Su et al., 2015).

## 5 Conclusion

We investigated whether PVC based on CT (obtained by PET/CT) can improve the evaluation accuracy of amyloid deposits by amyloid PET imaging. Our results strongly suggest the feasibility of PVC for amyloid PET based on CT. In addition, we found that the results of CT‐based PVC correlated with those of MRI‐based PVC, with some differences, indicating that CT may replace MRI for PVC, which is advantageous, given the popularity of PET/CT scanners. We conclude that with the proper methodology of PVC, using ^11^C‐PiB PET/CT will lead to efficient and reliable AD assessment.

## Funding Information

NCNP (Grant/Award Number: ‘Intramural Research Grant (27‐8) for Neurological’).

## Conflict of Interest

None declared.
